# FTIR, Raman and AFM characterization of the clinically valid biochemical parameters of the thrombi in acute ischemic stroke

**DOI:** 10.1038/s41598-019-51932-0

**Published:** 2019-10-29

**Authors:** Aneta Blat, Jakub Dybas, Karolina Chrabaszcz, Katarzyna Bulat, Agnieszka Jasztal, Magdalena Kaczmarska, Roman Pulyk, Tadeusz Popiela, Agnieszka Slowik, Kamilla Malek, Mateusz G. Adamski, Katarzyna M. Marzec

**Affiliations:** 1 Jagiellonian Center for Experimental Therapeutics, Jagiellonian University, 14 Bobrzynskiego Str., 30–348 Krakow, Poland; 2 Faculty of Chemistry, Jagiellonian University, 2 Gronostajowa Str., Krakow, Poland; 3 Center for Medical Genomics (OMICRON), Jagiellonian University Medical College, 7c Kopernika Str., 31–034 Krakow, Poland; 4 Department of Neurology, Jagiellonian University Medical College, 3 Botaniczna Str., 31–503 Krakow, Poland; 5 Department of Neuroradiology, Jagiellonian University Medical College, 3 Botaniczna Str., 31–503 Krakow, Poland

**Keywords:** Optical spectroscopy, Stroke, Infrared spectroscopy, Raman spectroscopy, Atomic force microscopy, Confocal microscopy, Lipids, Proteins, Imaging studies, Infrared spectroscopy, Medical and clinical diagnostics, Bioanalytical chemistry

## Abstract

The significance and utility of innovative imaging techniques in arterial clot analysis, which enable far more detailed and automated analysis compared to standard methods, are presented. The examination of two types of human thrombi is shown, representing the main ischemic stroke etiologies: fibrin–predominant clot of large vessel origin and red blood cells–rich clot of cardioembolic origin. The synergy effect of Fourier–transform infrared spectroscopy (FTIR), Raman spectroscopy (RS) and atomic force microscopy (AFM) techniques supported by chemometrics in comparison with reference histological staining was presented. The main advantage of such approach refers to free–label and non–destructive quantitative imaging of clinically valid, biochemical parameters in whole sample (FTIR–low resolution) and selected regions (RS–ultra–high resolution). We may include here analysis of lipid content, its distribution and total degree of unsaturation as well as analysis of protein content (mainly fibrin and hemoproteins). The AFM studies enhanced the vibrational data, showed clearly shape and thickness of clot features as well as visualized the fibrin framework. The extraordinary sensitivity of FTIR and RS imaging toward detection and discrimination of clinically valid parameters in clot confirms its applicability in assessment of thrombi origin.

## Introduction

Stroke is the fifth cause of death globally accounting 6.5 millions of deaths worldwide^1^. Ischemic stroke (IS), caused by vessel occlusion represents 87% of all stroke cases. Thrombectomy, with mechanical clot retriever, is one of the recanalization treatment options for IS. This technique also enables collecting and analyzing of the fresh thrombus samples. It has been recognized that the molecular analysis of the clot composition and its physical properties may provide information on selection of recanalization strategy and establishing of stroke etiology that are crucial for clinical outcome and secondary stroke prevention^2,3^. Thus ongoing studies on extracted thrombotic clots have focused on the analysis of their histology composition and have identified the presence of accumulated platelets, fibrin, red blood cells (RBCs) and white blood cells (WBCs)^4^. Computed tomography (CT) and magnetic resonance imaging (MRI) studies have been correlated previously with the clot histological composition to assess thrombolytic recanalization outcome and IS etiology. A lower clot density (in Hounsfield units, HU) was correlated with a higher resistance to the pharmacological lysis and mechanical thrombectomy^2^. In turn a high WBCs content in thrombi was associated with the cardioembolic etiology, extended mechanical recanalization time and less favorable recanalization and clinical outcome^5^. The RBCs–rich clots had higher density than platelet–rich thrombi^2^ and were more susceptible to intravenous and intra–arterial thrombolysis^6^. On contrary a number of studies have reported that CT and MRI give poor prognostic information on clinical outcome and have a limited clinical diagnostic value^4,7,8^. It is a heterogeneous disease with numerous underlying causes. Although the numbers of etiopathological classifications have been introduced, stroke etiology for significant number of patients cannot be defined. Published data indicated that an embolic source of stroke remains unidentified in 25–38% of patients while its origin is crucial for patient’s classification in clinical trials, epidemiological or genetic studies and most of all for therapeutic decision–making in everyday practice^9^.

Herein, we propose the combination of two vibrational spectroscopies (VS): Fourier–transform infrared spectroscopy (FTIR) and Raman spectroscopy (RS) supported with atomic force microscopy (AFM) and histological staining as a potential set of tools for the determination of acutely retrieved clot composition. We have previously described and reported the usefulness of such a set of techniques for the characterization of atherosclerotic plaque and other tissue alterations^10–13^. In the present work, we show that the applied techniques enable the detection, visualization and differentiation of various features based on their biochemical (VS) and physical properties (AFM).

**Figure 1:**
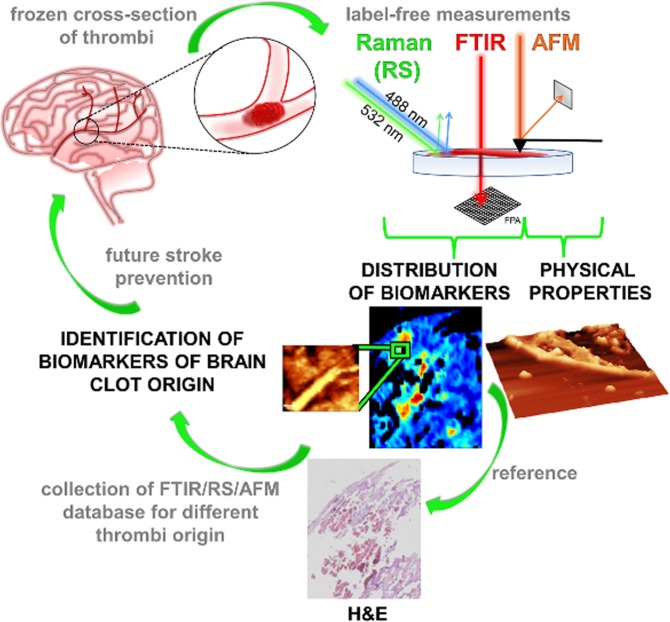
Schematic illustration of acute IS thrombi characterization with the application of FTIR, RS and AFM.

As presented in Fig. 1, the frozen cross–sections of the retrieved thrombi were firstly examined with free–label FTIR imaging which allowed us to study the distribution of various clot features with the spatial resolution of around 5.5 µm^14^. Further on, the free–label RS imaging technique was applied for the chosen regions of interest (ROI) to detect biochemical markers with the spatial resolution of around 0.4 µm^11,12^. At the end topographies of chosen ROIs were obtained with the use of AFM. Hematoxylin and eosin (H&E) staining was used as a reference. We investigated thrombi from different etiology of the acute ischemic stroke: a fibrin–predominant clot of a large vessel origin and RBCs–rich clot of a cardioembolic origin. The main goal of this work was to present the benefits of the combination of FTIR, RS and AFM techniques for the thrombi analysis and to highlight the potential of such assessment to identify brain clot origin – crucial for future stroke prevention. In addition, our report has a retrospective nature for the future development of the new and label–free VS imaging based method for the clot identification. Obviously, further VS investigations, combined with extensive histological examination on a statistically significant number of thrombi, are required to confirm presented applicability. To the best of our knowledge this is the first study to utilize label–free and chemically sensitive imaging of acute IS retrieved clot.

## Results

The results section is divided into two parts which presents the analysis of thrombi which were representative for a different etiology of the acute ischemic stroke: one sample was derived from a patient suffered from large vessel stroke due to artery dissection while two other samples were collected from two patients with cardioembolic stroke.

### Fibrin–predominant clot of a large vessel origin

#### FTIR–based chemical composition imaging combined with H&E staining

**Figure 2:**
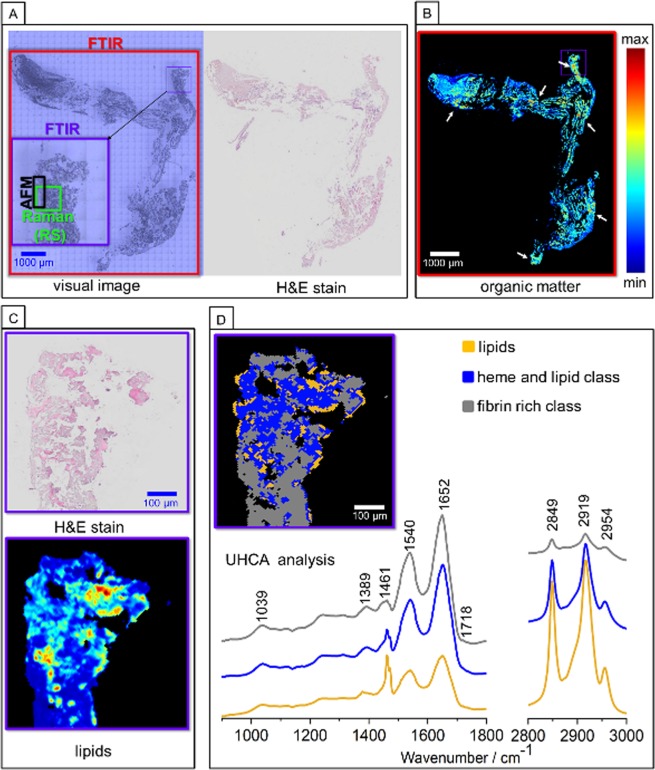
Representative FTIR imaging of human brain clot of a large vessel origin. (**A**) White–light optical image (20×) with marked ROIs for FTIR (red, purple), RS (green) and AFM (black) imaging with H&E stained image; (**B**) FTIR spectral image of distribution of organic matter (integration in the 2800–3100 cm^−1^ region) with lipid–rich areas marked with white arrows; (**C**) H&E staining with the chosen ROI presented in (**B**), (**D**) the UHCA false–color map with mean FTIR spectra.

Figure 2A presents H&E staining of the cross–section of the whole extracted brain clot, showing its morphology and cellular heterogeneity. Different parts of thrombus reveal a diversity of histological pattern, wherein fibrin is stained in pink and is present as a homogenous area localized on the edges and in the middle of the clot. RBCs are stained in red, while cell nuclei in violet. H&E staining indicates the presence of a fibrin predominant clot type.

FTIR image of the organic matter distribution was constructed by integration of the 2800–3100 cm^−1^ region, which is especially intense for bands of the methylene and olefinic groups (Fig. 2B)^15–17^. This spectral region easily differentiates the clot areas rich in the lipid fraction due to the presence of long-chain fatty acids and also can be used for lipids quantification as shown previously^18^. We calculated lipid–to–clot area ratio in each imaged ROI, cf. Table SM1. Overall 13.19% of lipids fraction is present in the fibrin–predominant clot and they are not distributed homogeneously. As revealed by the comparison of the FTIR–based lipids distribution image with H&E stains presented in Fig. 2, the free lipid–rich classes are mainly surrounded by RBCs. In order to define the chemical variety of the observed lipid composition, we performed cluster analysis (CA) for chosen, smaller ROIs of the clot that are rich in lipid fraction marked with arrows in Fig. 2B. The analyses for all ROIs are presented in SM (Fig. SM1). An example of such approach is presented in Fig. 2D, where unsupervised hierarchical cluster analysis (UHCA) discriminated 3–different classes: fibrin, heme with lipid contribution and lipids^18–20^. These different components of the clot exhibit the spectral changes mainly in the regions associated with the symmetric (sym.) and asymmetric (asym.) stretching vibrations(ν) of CH_2_ groups [ν(CH_2_) modes] observed at 2849 and 2919 cm^−1^, respectively, and attributed mainly to the high content of lipids or biomolecules containing long acyl chains^21^. The detailed analysis of the lipid fraction is based on the comparison of the FTIR spectra with the standard compounds and is discussed in Sub–section 3.1.3. Fibrin– and heme–rich classes are characterized in FTIR spectroscopy by domination of intensive amide I at 1652 cm^−1^ and amide II at 1540 cm^−1^ bands^22^. More intense amide bands correspond to the protein–rich class containing fibrin. As we mentioned, RBCs are localized nearby lipids, hence contributions of band at 1461 cm^−1^ as well as intense bands located at 2849 and 2919 cm^−1^ in the heme class are expected^18^.

#### Chemical analysis of the clot with the use of RS and AFM supported with H&E

**Figure 3:**
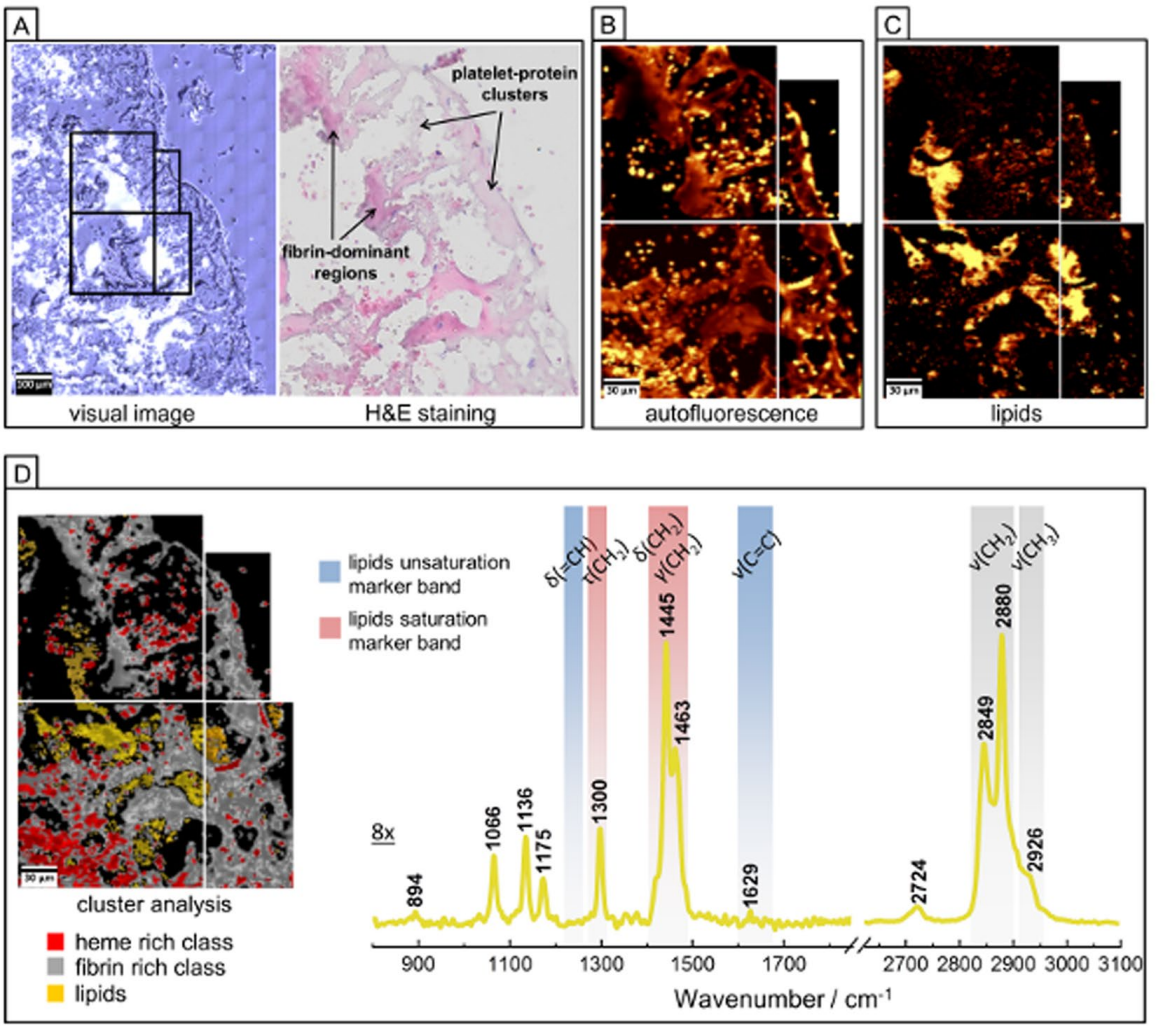
RS imaging of human brain clot of a large vessel origin. (**A**) White–light optical image (100×) from ROI presented in Fig. 2A with H&E staining; (**B**) Auto–fluorescence image (integration in the 2100–2500 cm^−1^ range); (**C**) RS image of lipids distribution (integration in the 2830–2860 cm^−1^ range); (**D**) K–means CA image and average RS spectra of the lipid–rich class. The spectral range of 800–1850 cm^−1^ was multiplied by factor 8 comparing to higher region for clarity. RS spectra were collected with the use of the 532 nm laser excitation.

Figure 3 presents the exemplary ROI of the clot selected for RS imaging (marked in green in Fig. 2A) and its magnified H&E microphotograph with identified fibrin–dominant regions (intense pink color), platelet–protein clusters (pale pink color) as well as few areas with cell nuclei (violet–colored dots). The auto-fluorescence of specific areas of cells and tissues can be studied in parallel to the Raman signal collection^23^ (for a specific laser wavelength) and it is manifested as an increased background signal^24^. For example this phenomena was successfully applied for visualization of elastic lamina or fibrous caps in atherosclerotic plaques^10,13^. On the other hand, the increase of auto-fluorescence can be assigned to RBCs, where the closely packed heme groups of hemoglobin absorb a considerable amount of Vis photons. This process causes an additional generation of photothermal effects leading to raised background in Raman spectra of RBCs^25–27^. This enabled us to assume that auto–fluorescence image displayed in Fig. 3 revealed the localization of the dried RBCs (bright yellow spots in Fig. 3B). Pixel spectra characterized by the less intense auto-fluorescence region indicated the presence of fibrin (darker shade areas in Fig. 3B, spectra presented in Fig. SM2)^10,13^. Raman integration image in the region of 2830–2860 cm^−1^ revealed the localization of the lipid–rich grease class. K–means CA distinguished three classes which correspond to the areas of the clot rich in fibrin, heme and lipids (Fig. 3D). The averaged lipid spectrum obtained from CA is dominated by modes located at 2849 and 2880 cm^−1^ originated from the sym. and asym. ν(CH_2_) modes, respectively^12,28^. Moreover, RS technique can easily provide the information about the general lipid unsaturation level. The total degree of unsaturation of the grease lipid–rich class of the clot can be calculated on the base of the RS intensity ratio of the bands at around 1266/1300 cm^−1^ or at 1655/1444 cm^–1 ^^28^. In the case of the presented average RS spectrum of the lipid–rich class of clot, the bands at around 1266 and 1655 cm^−1^, connected with the bending vibration of =CH group and ν(C=C) modes, respectively, are almost invisible, what clearly indicates the absence of unsaturated lipids. Moreover, presented previously Raman database of lipids^28^, suggests presence of lipids mixture, which detailed analysis is presented in chapter 3.1.3.

**Figure 4:**
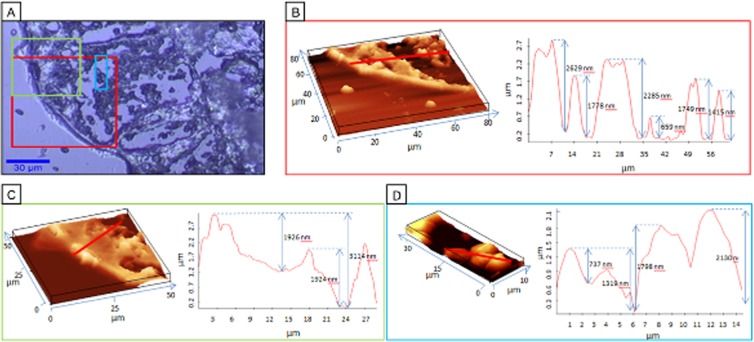
AFM imaging of human brain clot of a large vessel origin. (**A**) White–light optical image (100×) of the cross section of fibrin–predominant clot (marked in Fig. 2A) with the investigated ROIs labeled with red, green and blue; (**B–D**) the AFM topography images in 3D view and topography profile according to red line for presented in A ROIs.

Figure 4 presents three ROIs of the clot, marked by black rectangle in Fig. 2A which were chosen for AFM measurements. AFM is a very useful technique which can complement the VS data with topographical and mechanical information about the sample^10^. Here, the chosen ROIs were analyzed from the point of its shape and topography profiles in order to differentiate various clot structures. The fixed and frozen clot sample, was cut into 10 µm slices but due to structural differences between clot components they dried in different manner. Therefore, studying the topography variation of dried samples delivers information about different features, as presented previously^10^. The topography of the red and green ROIs clearly showed that some areas of the clot stick out from the plane what corresponds to the clot scaffolding. Such areas were assigned to the fibrin skeleton of the clot as H&E staining indicated. The edge is the highest and the stiffest in the entire body of the clot (Fig. 4B,C). The average difference in the height between outer fibrin skeleton and the internal fibrin skeleton reached 2–3 µm. A similar variation in the surface topography has been previously noticed for the fibrous cup observed on the surface of the atherosclerotic plaque^10^. AFM features of the red and blue ROIs indicated the presence of blood cells, platelets and lipids distributed unevenly between the fibrin patterns. The AFM results indicate that RBC, WBC and platelets stiff parameters are inferior in comparison to the fibrin skeleton parameters. As we previously reported^10^, it was also possible to differentiate the crystals of cholesterol from the different type lipid–rich grease areas.

#### Detailed analysis of lipids composition – FTIR and RS spectroscopies

**Figure 5:**
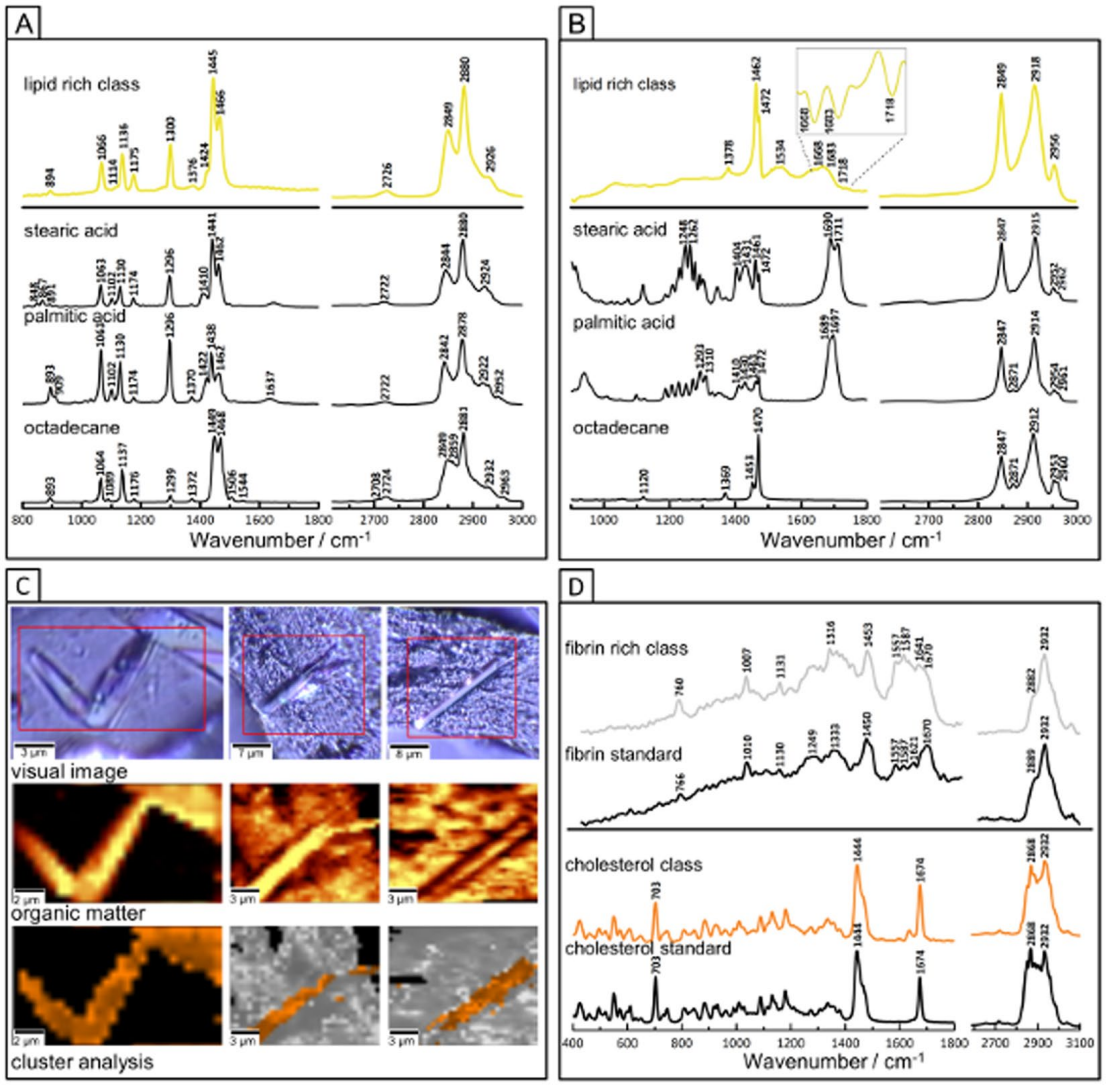
RS and FTIR spectra of lipids in human brain clot of a large vessel origin compared with spectra of standard compounds. (**A**) RS spectra (532 nm excitation) of the averaged lipid–rich grease class presented in Fig. 3 (yellow line) compared to the chosen lipid standards; (**B**) FTIR spectra of the averaged lipid–rich grease class presented in Fig. 2B compared to the chosen lipid standards; (**C**) RS CA images of the cholesterol crystals and fibrin–rich class; (**D**) the mean RS spectra of to the classes presented in C compared to standards of fibrin and cholesterol.

RS and FTIR spectra, extracted for the lipid–rich grease, are as were compared with the spectra of the standard compounds published so far of various carboxylic acids, triglycerides and alkanes with the different number of carbon atoms^18,28^. Figure 5 shows the comparison of the FTIR and RS spectra of the grease, lipid–rich class with selected, most similar reference long acyl–chain compounds, i.e. stearic acid, palmitic acid and octadecane. The intensity ratio of the sym. and asym. ν(CH_2_) modes of the lipid–rich grease is similar to the ratio observed in the RS spectra of palmitic and stearic acids whereas the position of the sym.ν(CH_2_) mode in the RS spectrum of lipid–rich class corresponds rather to the band of octadecane than fatty acids. Bands observed at 1445 and 1463 cm^−1^ are assigned to the γ(CH_2_) and δ(CH_2_) modes, respectively^28,29^. While bands at ca. 1300 cm^−1^ and in the region of 1200–1060 cm^−1^ originate from the twisting CH_2_ and ν(C–C) vibrations, respectively^28^. The spectral profile of all those modes is similar to the spectrum of stearic acid. However, the absence of the ν(C=O) vibrations at approximately 1640 cm^−1^, suggests carbohydrate component such as long–chain octadecane. Similarly, to RS, the FTIR spectrum of the lipid–rich clot class was compared to the FTIR spectra of the same set of long acyl chain compounds (Fig. 5B). Spectral similarities with the standards are connected mainly with the ratio for bands located at 2918 and 2849 cm^−1^ assigned to the sym. and asym. ν(CH_2_) vibrations, respectively. FTIR spectrum extracted from lipid–rich class is characterised by the presence of the band at 1378 cm^−1^, 1462 cm^−1^ and 1472 cm^−1^ assigned to sym. δ(CH_3_), asym. δ(CH_2_) and γ(CH_2_) modes, respectively. The amide I (1653 cm^−1^) and amide II (1543 cm^−1^) bands observed in the lipid–rich spectrum originate rather from other tissue components in this class (proteins). The absence of ν(C=O) modes approximately at 1710–1690 cm^−1^, suggests that the lipid–rich class of the clot contribute from the long–chain alkane. However, the ratio of bands at 1462 and 1472 cm^−1^ and the visible only in the second derivative spectrum, very weak vibrations from C=O groups, suggest also the presence of the carboxylic acids.

Both, FTIR and RS spectroscopies suggests that the lipid–rich grease areas in the clot is a mixture of different chemical with the majority fraction consisting of long chain saturated fatty acids (SFA) and alkanes with around 16–18 atoms of carbon. Such presence of alkanes next to fatty acids produced due to the oxidative stress is not surprising and plays a crucial role in IS pathogenesis^30^. Additionally, the high resolution (hi-res) RS spectroscopic imaging indicated the presence of cholesterol in a form of few–micrometric crystals present in the clot structure (Fig. 5C,D). The most prominent bands of cholesterol are located at 703, 1443 and 1674 cm^−1^ and are assigned to the sequence to the cholesterol backbone vibrations such as the γ(CH_2_) and ν(C=C) modes of the cholesterol ring^10,28^. In the high–wave number region, a very typical doublet at 2868 and 2932 cm^−1^ originated from CH_2_ modes is observed^10,28^.

### RBCs–rich clot of cardioembolic origin

**Figure 6:**
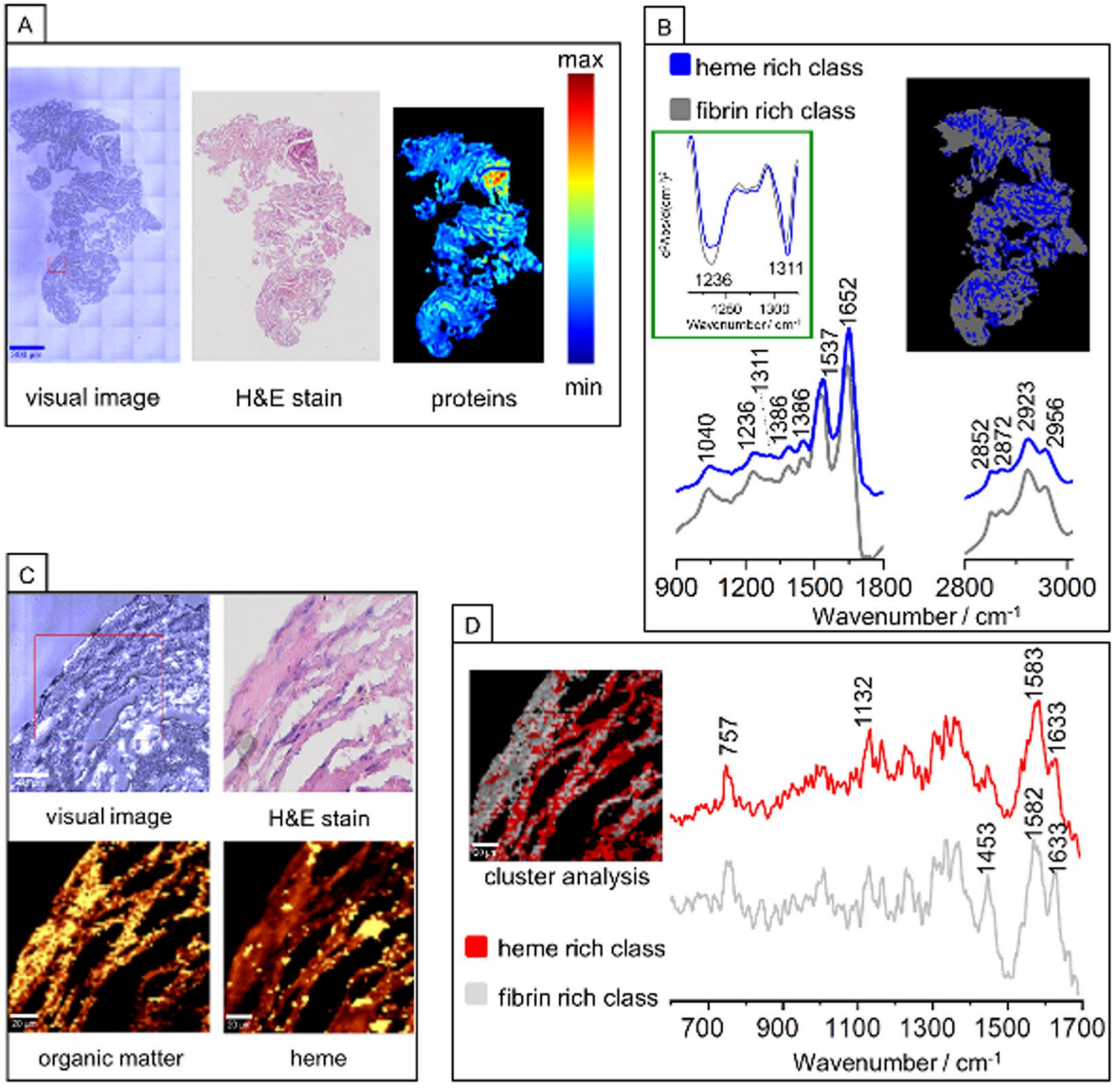
FTIR and Raman imaging of a RBCs–rich human brain clot of cardioembolic origin. (**A**) White–light optical image (20×), H&E staining and FTIR image of proteins distribution; (**B**) UHCA image, (performed in regions of 914–1800 and 2800–3100 cm^−1^) with its average second derivative FTIR spectra corresponding to heme–rich class and fibrin–rich class; (**C**) White–light optical image (x100), H&E staining and Raman integration images (532 nm excitation) of the organic matter (integration in the 2800–3050 cm^−1^ range) and heme (integration for the 757 cm^−1^); (**D**) CA image with average Raman spectra for heme–rich class and fibrin–rich class.

Figures 6 and SM3 illustrate H&E staining and FTIR integration images of protein distribution for the chosen area of RBCs–rich human brain clot of cardioembolic origin. The comparison of H&E staining with FTIR UHCA analysis allowed us to distinguish two main biological features of the heme–rich class and fibrin–rich class. Domination of these protein structures in clot was confirmed by appearance of the band at 2872 cm^−1^ assigned to sym. ν(CH_3_) mode and high intensities of amide I and amide II bands^22^. In the region of 1324–1200 cm^−1^ attributed to the amide III range we identified two bands at around 1311 and 1236 cm^−1^ corresponding to α–helix and β–sheet secondary structure, respectively^31^. Based on the comparison of intensities of the bands at 1236 cm^−1^ and 1311 cm^−1^ we were able to assign recorded spectra to fibrin and heme, respectively. The grey spectrum with the higher intensity of β–sheet band in comparison to α–helix band was attributed to fibrin class. It is known, that fibrin consists of the mixture of α–helices, β–sheets and other secondary structures and we explain this assignment by the α–helix to β–sheet transition as a result of the formation of clot in dynamic conditions (flowing blood)^19,32^. The blue class was relegated to heme structure of RBCs.

Figure 6C,D present the magnification of the H&E staining for the chosen ROI together with Raman integration images based on the organic matter region, heme rich region and CA images with differentiation of two classes corresponding to the areas rich in fibrin and heme. As revealed by the averaged RS spectra obtained from CA analysis both, heme– and fibrin–rich classes are composed of similar modes with bands characteristic for porphyrins and non–heme proteins, respectively. The fibrin–rich spectrum may be however differentiated from heme–rich spectrum by observation of the intensity ratio of Amide I mode (1633 cm^−1^) to 1584 cm^−1^ mode originating from C–C porphyrin in–plane vibrations. This RS distribution is consistent with the UHCA image constructed from FTIR data.

## Discussion

This work demonstrates the significance and utility of free–label FTIR, RS and AFM techniques in arterial clot analysis in comparison with reference histological staining. Synergy effect of these free–label imaging techniques, delivering vast amount of sample data, enables far more detailed analysis compared to standard methods. This is demonstrated based on the examination of two types of thrombi, representing main IS etiologies: fibrin–predominant clot of large vessel origin and RBCs–rich clot of cardioembolic origin; where wide spectrum of clinically valid parameters is assessed with FTIR, RS and AFM techniques. Table 1 summarizes studied thrombi features in respect to each imaging technique capabilities.

**Table 1:** The comparison of the analytical potential of FTIR, RS and AFM in the thrombi studies.

Thrombi feature	FTIR	RS	AFM	References
lipid content	• distribution within the whole cross–section • qualitative or semi–quantitative detection	• distribution with hi–res for chosen ROI • qualitative measurement	• form of lipids (crystals/grease material)	^10, 18, 23^
the total degree of lipids unsaturation	• semi–quantitative measurement for whole cross–section	• semi–quantitative measurement with hi–res for chosen ROI	–	^38, 39^
general proteins content	• distribution for whole cross–section • qualitative or quantitative measurement	• distribution with hi–res for chosen ROI • qualitative or semi–quantitative measurement	• height and topography/stiffness of tissue	^14^
the ratio of lipids to proteins	• semi–quantitative measurement for whole cross–section	• semi–quantitative measurement with hi–res for chosen ROI	–	^11, 12^
fibrin content	• distribution for whole cross–section • qualitative measurement • in order to build the model necessary to compare with H&E	• distribution with hi–res for chosen ROI • qualitative measurement • in order to build the model necessary to compare with H&E	• fibrin height and topography/stiffness	^10, 13, 40, 41^
RBCs/heme	• distribution for whole cross–section • qualitative measurement	• distribution with hi–res for chosen ROI • qualitative measurement • high specificity and sensitivity to heme due to resonance effect	• height and topography/stiffness	^42, 43^

The main advantages of the VS application, both FTIR and RS, over standard methods of clot composition analyses, refer to label–free and non–destructive quantitative imaging of chosen biochemical compound in whole sample (FTIR–low resolution) and selected region (RS–ultra–high resolution). In addition to the analyses conducted within this study applied techniques enable qualitative and semi–quantitative assessment of type of lipids with their distribution, define the total degree of lipids unsaturation, present the ratio of total lipids to total proteins, differentiate the heme/RBCs from fibrin content. Capabilities of FTIR and RS make it a very flexible research tool for thrombus analysis.

The FTIR and RS analyses of the fibrin–predominant thrombus obtained from a person with aortic dissection enable discrimination of three molecular classes: fibrin, heme and different types of lipids. The detailed analysis of RS and FTIR spectra obtained from lipid–rich grease class allowed to assign these clot lipids to the mixture of SFA and alkanes with 16–18 atoms of carbon, which are known to play a crucial role in ischemic stroke. FTIR additionally allowed calculation of lipid–to–clot area ratio concluding that overall 13.19% of lipids fraction is present in the fibrin–predominant clot and revealed non–homogeneous distribution. RS allowed us to calculate the total degree of lipid unsaturation and indicated the saturated lipid dominance. Additionally, the high resolution RS spectroscopic imaging revealed the presence of cholesterol in a form of few–micrometric crystals in clot structure. The morphology of cholesterol crystals and grease lipid–rich class was confirmed by AFM. The H&E staining was far more time–consuming, destructive for sample in terms of lipids rinsing due to requirement of sample dehydration and finally did not allow for lipids visualization. Therefore, this method is not suitable for the lipids detection but is useful as clot morphology reference. The AFM studies enhanced the vibrational data and showed clearly shape and thickness of the clot sample. The AFM results revealed that fibrin framework creates skeleton for the whole thrombus structure. The RS as well as the FTIR integration images of the RBCs–rich clot enabled to distinguish two different protein structures of heme of RBCs and fibrin. The RBCs–rich clot obtained from the patient with the cardioembolic origin was found to not contain lipid fraction.

Presented results, based on three cases, indicate potential diagnostic role of VS in identification of thrombi origin. First thrombus of large vessel origin, identified as fibrin–predominant, was obtained from the patient with aortic dissection. It was rich in different types of lipid fractions, including mainly SFA. This finding supports vascular origin of thrombotic material as lipids are commonly present in inflammatory changed vessel wall^33^. Furthermore, SFA are common risk factor for vascular diseases^34^. Remaining two thrombi, identified as RBC predominant, were both obtained from patients suffering from cardioembolic stroke. As expected, lipids were not detected and their main components were heme from hemoglobin and fibrin.

Sample size of this study does not allow drawing clinical conclusions neither on pathomechanisms nor stroke etiology. Presented results aimed to demonstrate added value of IR, RS and AFM techniques to future large scale clinical studies on IS. Such studies have a retrospective nature and will allow for a future development of the new, label–free imaging techniques for clot evaluation with the application of the VS.

## Conclusions

Application of two vibrational imaging techniques combined with AFM allowed for detection and visualization of various lipid content, RBCs accumulation and fibrin–rich areas in the clots, lipid differentiation and assessment of total degree of lipids unsaturation, semi–qualitative evaluation of total number of lipids as well as physical evaluation of the clot. Such methodology is complementary to the conventional histological staining, but is label–free, operator objective and automated. We proved that the VS can be the potential label–free set of tools for the thrombus composition evaluation. To the best of our knowledge this is the first work where the human brain clots were chemically characterized and visualized on the base of the FTIR and RS measurements, supported by AFM in combination with H&E.

## Methods

The study was approved by the Ethical Committee of the Jagiellonian University and all the experiments were performed in accordance with Ethical Committee of the Jagiellonian University guidelines and regulations. All patients and/or authorized representatives gave full and signed informed consent. A detailed description of materials and methods are presented in the supplemental materials (SM).

Three acute IS patients (N = 3), following informed consent, were included in this study. Each patient underwent mechanical thrombectomy with SOLITAIRE (Medtronic, USA) stent retriever; treatment was performed according to manufacturer protocol. Two patients suffered from cardioembolic stroke, one from large vessel stroke due to artery dissection^35^. Stroke type was classified to one of five etiologies: large vessel, small vessel disease, cardioembolic or unknown etiology, according to TOAST criteria^36^. From each fixed and frozen in optimal cutting temperature compound (OCT) clots, minimum three cross sections were cut and transferred onto CaF_2_ slides and then investigated by FTIR, RS and AFM spectroscopy. Finally samples were stained with hematoxylin and eosin (H&E) what requires dehydration with the use of concentrated alcohol^37^ and often results in rinsing of the lipids from the sample.

## Supplementary information

Supplementary information
