# Supplementary material for: FTIR, Raman and AFM characterization of the clinically valid biochemical parameters of the thrombi in acute ischemic stroke
Source: Sci Rep. 2019 Oct 29;9:15475. doi: 10.1038/s41598-019-51932-0 (PMC6820737; doi:10.1038/s41598-019-51932-0)
Supplement: Supplementary file 1 — Supplementary information [file 41598_2019_51932_MOESM1_ESM.docx]

**Supplemental Material**

**Title: FTIR, Raman and AFM characterization of the clinically valid biochemical parameters of the thrombi in acute ischemic stroke**

Aneta Blat^a,b^, Jakub Dybas^a^, Karolina Chrabaszcz^a,b,c^, Katarzyna Bulat^a^, Agnieszka Jasztal^a^, Magdalena Kaczmarska^a^, Roman Pulyk^d^, Tadeusz Popiela^e^, Agnieszka Slowik^d^, Kamilla Malek^b^, Mateusz G. Adamski^a^, Katarzyna M. Marzec^a,c*^

*^a^JagiellonianCenter for Experimental Therapeutics, Jagiellonian University, 14 Bobrzynskiego Str., 30–348 Krakow, Poland*

*^b^Faculty of Chemistry, Jagiellonian University, 2 Gronostajowa Str., Krakow, Poland*

*^c^Center for Medical Genomics (OMICRON), Jagiellonian University Medical College, 7c Kopernika Str., 31–034 Krakow, Poland*

*^d^Department of Neurology, Jagiellonian University Medical College, 3 Botaniczna Str., 31–503 Krakow, Poland*

*^e^Department of Neuroradiology, Jagiellonian University Medical College, 3 Botaniczna Str., 31–503 Krakow. Poland*

**Materials and Methods**

**5.1. Sample preparation and histopathological staining**

Three acute ischemic stroke (IS) patients, following informed consent, were included in this study. Each patient underwent mechanical thrombectomy with SOLITAIRE (Medtronic, USA) stent retriever, treatment was performed according to manufacturer protocol. Two patient suffered from stroke of cardioembolic etiology and one from stroke due to a large vessel disease.^1^ IS subtype was classified according to TOAST criteria.^2^ Subsequent to retrieval, clot samples were fixed in 4% buffered formalin, embedded in OCT medium, frozen in –80° C and cut into 10 µm slices. In order to avoid sample alteration caused by paraffin embedding, frozen samples were studied. It was previously reported that paraffin is known to affect the examination of lipid structure and content ^3–5^ which could be a biomarker of brain clot origin^6,7^. From each fixed and frozen in OCT clots, minimum three cross sections were cut and transferred onto CaF_2_ slides and then investigated by FTIR, Raman and AFM spectroscopy. Subsequently samples were stained with hematoxylin and eosin (H&E). Sample processing for H&E staining requires dehydration with the use of concentrated alcohol ^8^ what often results in rinsing of the lipids from the sample.

**5.2. FTIR spectroscopic imaging**

FTIR spectroscopy images were recorded in transmission mode with an Agilent 620–IR microscope and 670–IR spectrometer with a liquid nitrogen cooled MCT FPA detector comprising 16 384 pixels in a 128 × 128 grid format. Measurements were recorded with a 15× Cassegrain objective (NA=0.62) collecting 128 scans. All spectra were acquired in the range of 900 – 3800 cm^–1^ with spectral resolution of 4 cm^–1^. The area measured was ca. 700 × 700 µm^2^ (projected pixel size of 5.5 × 5.5 µm^2^).

Spectral pre–processing and chemometric analysis of FTIR images were performed with a CytoSpec software (ver. 2.00.01)^9^. A MatLab (R2015a) software was used for water vapor removal, if necessary. Prior to any analysis, the quality of each pixel–spectrum was evaluated using the sample thickness criterion according to intensity of the amide I band (1620 – 1680 cm^‒1^). The baseline correction with polynomial fitting was applied with polynomial of order 3 and numbers of baseline points set on 7. For all spectra, second derivatives were calculated using a Savitzky‒Golay algorithm and 9 smoothing points. Then unsupervised hierarchical cluster analysis (UHCA) with a Ward’s algorithm was performed in the regions of 914 – 1800 cm^‒1^ and 2800 – 3100 cm^‒1^, while the spectral distances were computed as D–Values. Mean spectra were extracted for further analyses.

**5.3. Raman spectroscopic imaging (RS) and atomic force microscopy (AFM)**

Raman and AFM measurements were performed using a WITec confocal CRM alpha 300 Raman microscope combined with an atomic force microscope. The spectrometer was equipped with a CCD detector (Andor) cooled to –60°C and a dry air Olympus MPlan FL N 100×/0.9 objective. Raman spectra from clot were recorded using a 488 nm solid state laser (laser power: ca.10 mW) and a 532 nm Nd:YAG laser (laser power: ca. 15 mW). The lasers were coupled to the microscope via an optical fibre with a diameter of 50 µm and the scattered light was directed to UHTS 300 spectrograph (with 600 grooves/mm grating, BLZ = 500 nm). The spectrograph was calibrated using radiation from a xenon lamp (WITec UV light source). In addition, the standard alignment procedure (single–point calibration) was performed before each measurement with the use of the Raman scattering line produced by a silicon plate (520.5 cm^–1^). The integration time for a single spectrum varied from 0.5 to 1 s. The spectral resolution was equal to 3 cm^–1^. Raman measurements and data analysis were performed using a WITec software (WITec Project Plus). All Raman maps based on integration of marker bands were obtained from the band area above the background without any spectral pre–processing. k–Means Cluster Analysis (CA) was performed after cosmic spike removal and background subtraction in the Manhattan–distance formulation. All Raman spectra presented in this work were additionally post–processed by smoothing (with 9 points).

All Raman images calculated by integration of specific bands were obtained without any spectral pre-processing. Integral intensities of selected bands were computed, drawing the straight line between a point of the wavenumber, in which the peak appears and a point of the wavenumber, in which the peak disappears. The area below this line was integrated. The auto–fluorescence images were constructed based on background increase measured as an integral integration of the 2100 – 2500 cm^-1^ region which is typically Raman silent for biological samples.^10^

The AFM studies were carried out in the PFM mode (Pulsed Force Mode) using standard force modulation probes (WITec, Ulm, Germany) with a nominal spring constant of 2.8 N/m. A dry objective of Zeiss EC EPIPLAN 20×/0.4 was used. AFM images of 256 × 256 lines were collected from an area of 80 × 80 µm^2^ in Figure 4B, 50 × 50 µm^2^ in Figure 4C and 10 × 30 µm^2^ in Figure 4D. AFM data analysis was performed using a WITec software (WITec Project Plus) (topography cross sections) and Gwyddion 2.50 (correct horizontal scars (strokes), 3D view of data).


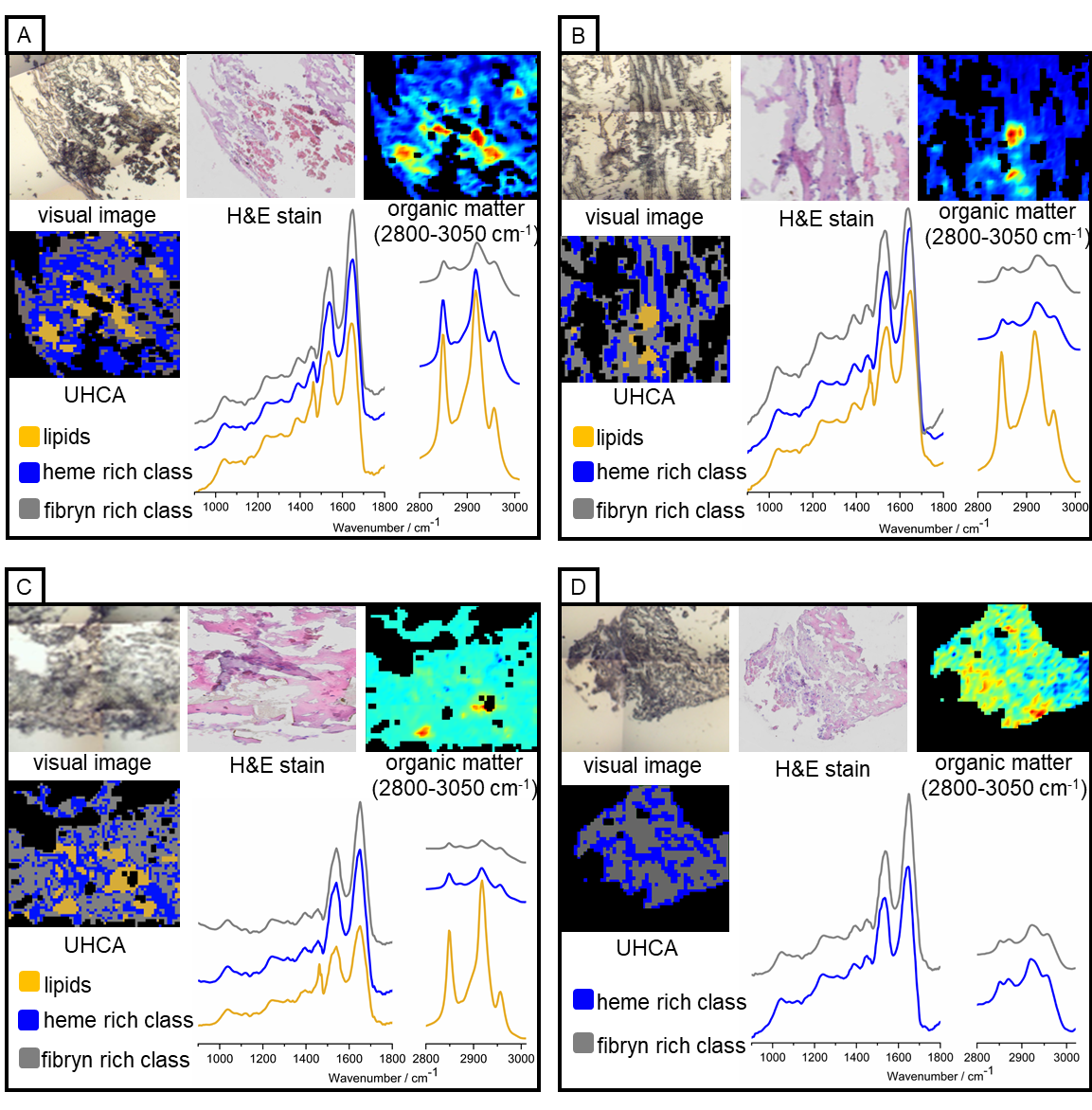
**Figure SM 1.FTIR imaging of large vessel origin clot retrieved from the human cerebral artery (patient 1).**White–light images of chosen ROIs (A, B, C, D) of human cerebral artery clot marked with white arrows in Fig. 2B (main text of manuscript) studied by FTIR imaging with H&E staining, IR spectral images presenting distribution of lipids (integration in the 2800 – 3100 cm^–1^ range) and the corresponding unsupervised hierarchical cluster analysis (UHCA, performed in the regions of 914 – 1800 and 2800 – 3100 cm^–1^). Mean FTIR spectra calculated by UHCA analysis; the colors correspond to the following classes: yellow – a lipid–rich class; grey – a fibrin–rich class; blue – a heme–rich class.


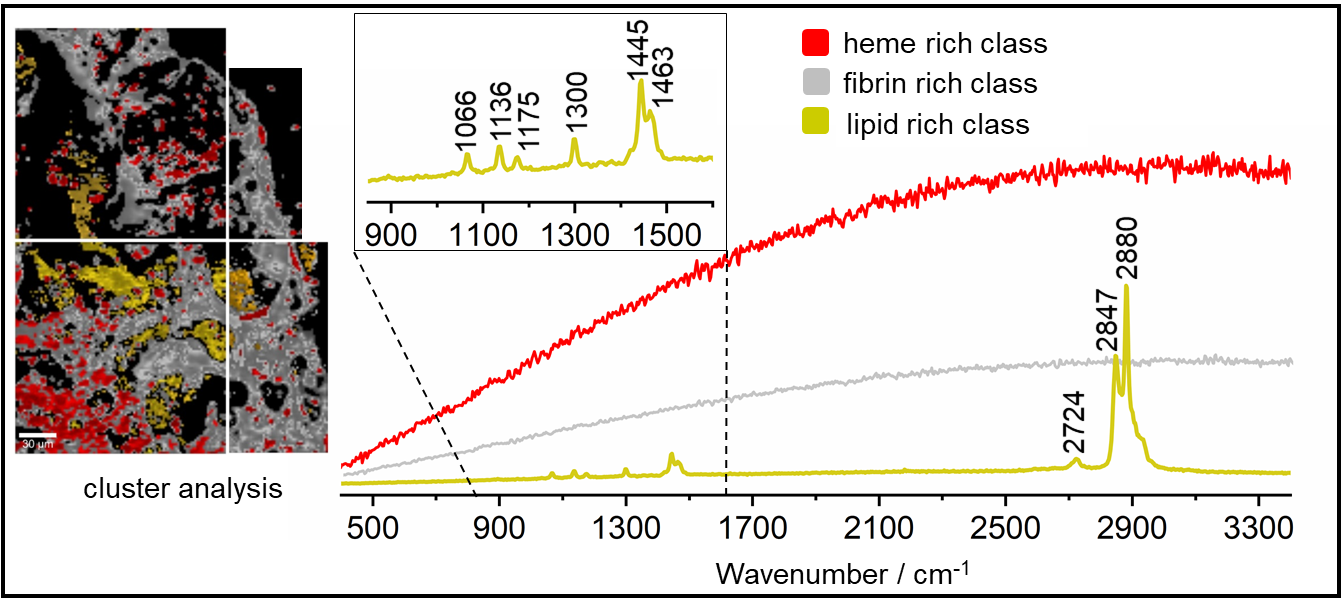


**Figure SM 2. Raman imaging of the large vessel origin clot retrieved from the human cerebral artery (patient 1).** Cluster analysis (CA) image and average Raman spectra taken from CA results with colors corresponding to CA classes as follows: red – heme–rich class; grey – fibrin–rich class; yellow – lipids. The insert shows enlarged range (850 – 1600 cm^–1^) of the Raman spectrum for lipids. All the Raman data was obtained with the use of 532 nm laser excitation.


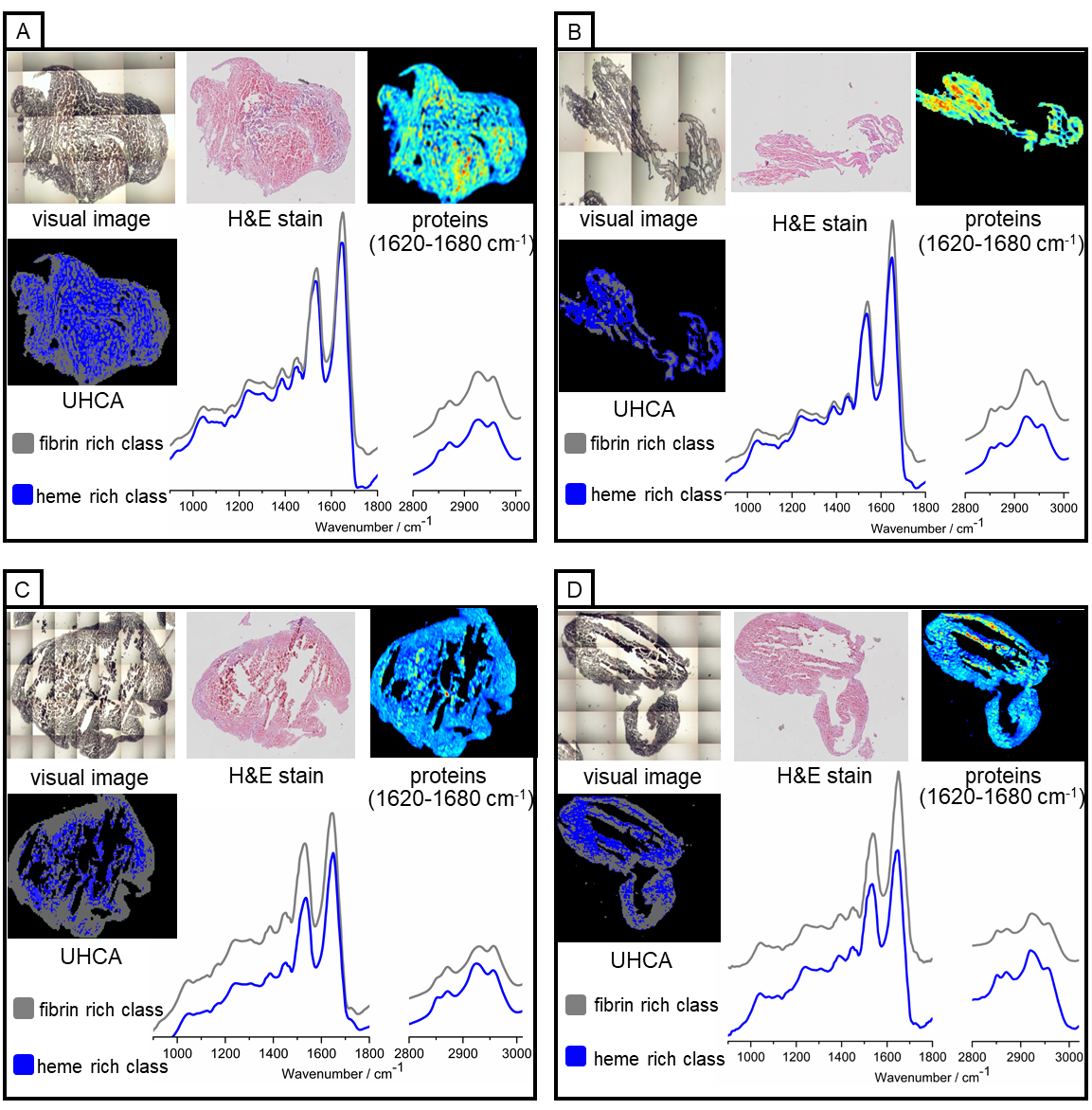


**Figure SM 3. FTIR and Raman imaging of a RBCs–rich cardioembolic origin clot retrieved from the human cerebral artery (patient 3).** Visual image of chosen areas (A, B, C, D) of human cerebral artery fibrin clot studied by FTIR imaging with H&E staining of the cross–section of human cerebral artery clot of cardioembolic origin, IR spectral image presenting distribution of proteins (integration in the 914 – 1800 cm^–1^ range) and corresponding unsupervised hierarchical cluster analysis (UHCA, performed in regions of 914 – 1800 and 2800 – 3100 cm^–1^). Average IR spectra were taken from UHCA map, colors corresponding with classes: grey – fibrin–rich class; blue – heme–rich class.

**Table SM 1.** IR images (1A–1K) present distribution of fibrin–predominant clot organic matter, proteins (1620–1680 cm^–1^); 2A–2F reveal distribution of lipids (2800–3100 cm^–1^) in those areas. In some imaged mosaics (1G–1K) no lipid influence were discovered.


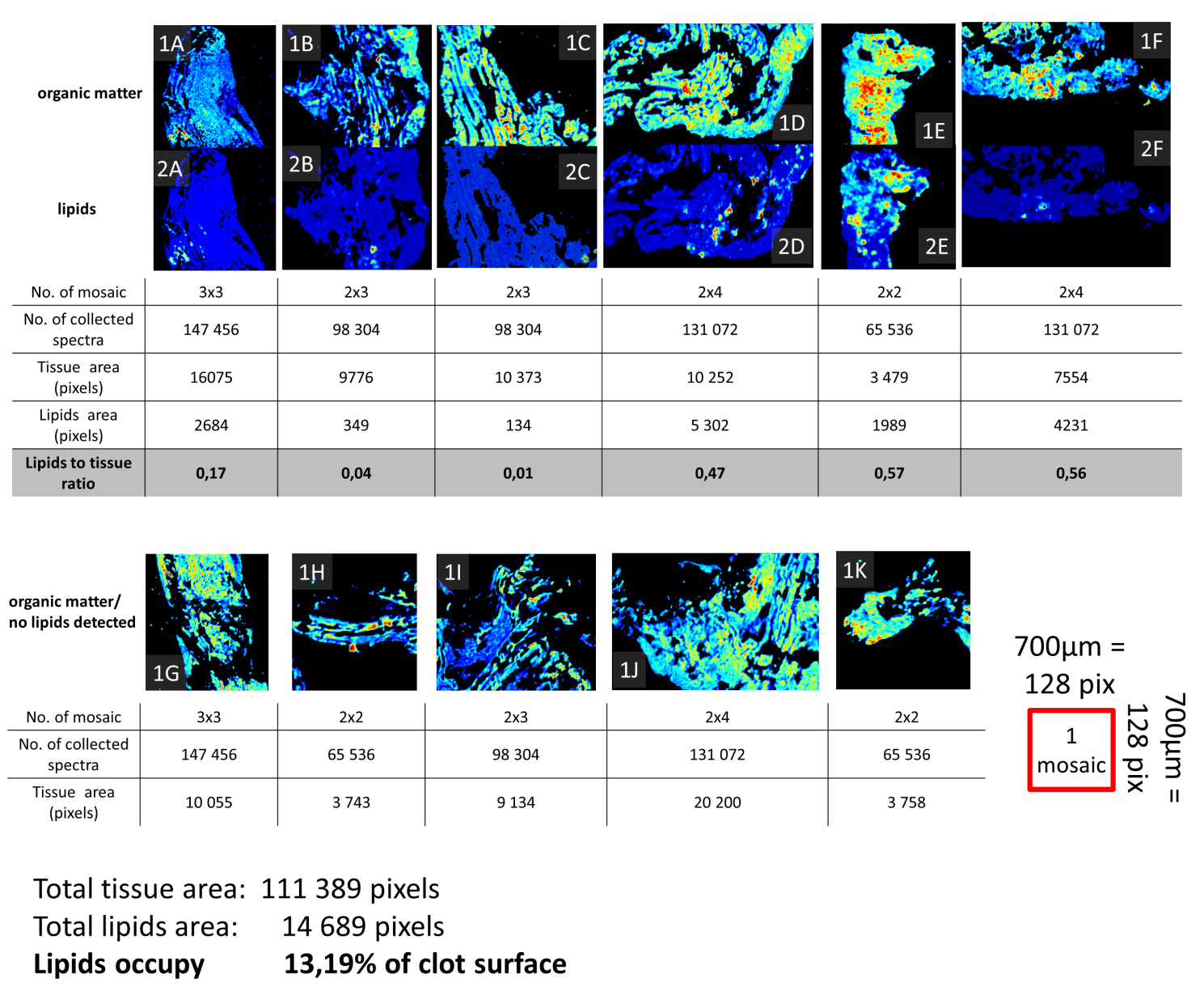


**SM References**

1. Adams H., Adams H., Bendixen B., Bendixen B., Kappelle L., Kappelle L., et al. Classification of Subtype of Acute Ischemic Stroke. *Stroke*. 1993;23:35–41.

2. Kolominsky-Rabas PL, Weber M, Gefeller O, Neundoerfer B, Heuschmann PU. Epidemiology of Ischemic Stroke Subtypes. *Stroke*. 2001;32:2735–2740.

3. K. Chrabaszcz, K. Kochan, A. Fedorowicz, A. Jasztal, E. Buczek, L.S. Leslie, R. Bhargava, K. Malek, S. Chlopicki, K. Marzec. FT-IR- and Raman-based biochemical profiling of the early stage of pulmonary metastasis of breast cancer in mice. *Analyst*. 2018;143:2042–2050.

4. Kochan K, Marzec KM, Maslak E, Chlopicki S, Baranska M. Raman spectroscopic studies of vitamin A content in the liver: a biomarker of healthy liver. *Analyst*. 2015;140:2074–2079.

5. Marzec KM, Kochan K, Fedorowicz A, Jasztal A, Chruszcz-Lipska K, Dobrowolski JC, et al. Raman microimaging of murine lungs: insight into the vitamin A content. *Analyst*. 2015;140:2171–2177.

6. Adibhatla RM, Hatcher JF, Muralikrishna R, Hatcher JF. Role Of Lipids In Brain Injury And Diseases Role of lipids in brain injury and diseases. *Future Lipidol.* 2007;2:403–422.

7. Pathophysiology and Biomarkers in Acute Ischemic Stroke - A Review. *Trop. J. Pharm. Res.* 2013;12:1097–1105.

8. Fischer AH, Jacobson KA, Rose J, Zeller R. Hematoxylin and eosin staining of tissue and cell sections. *CSH Protoc.* 2008;2008:pdb.prot4986.

9. Lasch P. CytospecTM. A Matlab based application for infrared imaging.

10. Palonpon AF, Sodeoka M, Fujita K. Molecular imaging of live cells by Raman microscopy. *Curr. Opin. Chem. Biol.* 2013;17:708–715. Available from: http://dx.doi.org/10.1016/j.cbpa.2013.05.021
